# Transcriptomic Profile of Oral Cancer Lesions: A Proof-of-Concept Pilot Study of FFPE Tissue Sections

**DOI:** 10.3390/ijms26136263

**Published:** 2025-06-28

**Authors:** Madison E. Richards, Micaela F. Beckman, Ernesto Martinez Duarte, Joel J. Napenas, Michael T. Brennan, Farah Bahrani Mougeot, Jean-Luc C. Mougeot

**Affiliations:** 1 Department of Oral Medicine/Oral and Maxillofacial Surgery, Atrium Health Carolinas Medical Center, Charlotte, NC 28203, USA; madison.richards@atriumhealth.org (M.E.R.); joel.napenas@atriumhealth.org (J.J.N.); jean-luc.mougeot@atriumhealth.org (J.-L.C.M.); 2 Translational Research Laboratories, Cannon Research Center & Oral Medicine, Atrium Health Carolinas Medical Center, Charlotte, NC 28203, USA; micaela.beckman@atriumhealth.org; 3 Carolinas Pathology Group, Atrium Health Carolinas Medical Center, Charlotte, NC 28203, USA; ernesto.martinezduarte@atriumhealth.org; 4 Department of Otolaryngology/Head and Neck Surgery, Wake Forest University School of Medicine, Winston-Salem, NC 27101, USA

**Keywords:** OPMD, FFPE, RNAseq, OSCC, gene expression

## Abstract

Oral squamous cell carcinoma (OSCC) is a malignancy that affects the oral mucosa and is characterized by indurated oral lesions. The RNAseq of formalin-fixed, paraffin-embedded (FFPE) samples is readily available in clinical settings. Such samples have long-term preservation and can provide highly accurate transcriptomic information regarding gene fusions, isoforms, and allele-specific expression. We determined differentially expressed genes using the transcriptomic profiles of oral potentially malignant disorder (OPMD) FFPE oral lesion samples of patients who developed OSCC over years. A technical comparison was completed comparing breast cancer (BC) FFPE publicly available data in this proof-of-concept pilot study. OSCC FFPE samples were collected from patients (*N* = 3) who developed OSCC 3 to 5 years following OPMD diagnosis (*n* = 3) and were analyzed using RNAseq. RNAseq sequences from the FFPE OSCC samples and publicly available FFPE samples of BC patients (*n* = 6) (Gene Expression Omnibus Database, GSE58135) aligned to human reference (GRCh38.p13). Genes were counted using the Spliced Transcripts Alignment to a Reference (STARv2.7.9a) software. Differential expression was determined in R using DESeq2v1.40.2 comparing OSCC to BC samples. Principal component analysis (PCA) plots were completed. Differential Kyoto Encyclopedia of Genes and Genomes (KEGG) pathways were determined via the Pathviewv.1.40.0 program. STRING v12.0 was used to determine protein–protein interactions between genes represented in more than one KEGG pathway. STARv2.7.9a identified 27,237 and 30,343 genes among the OSCC and BC groups, respectively. DESeq2v1.40.2 determined 9194 differentially expressed genes (DEPs), 4466 being upregulated (OSCC > BC) and 4728 being downregulated (BC > OSCC) (padj < 0.05). Most significant genes included *KRT6B*, *SERPINB5*, and *DSC3* (5- to 10-fold change range; padj < 10 × 10−100). PCA showed that BC and OSCC samples clustered as separate groups. Pathviewv.1.40.0 identified 17 downregulated KEGG pathways in OSCC compared to the BC group. No upregulated KEGG pathways were identified. STRINGv12.0 determined Gene Ontology Biological Process enrichments for leukocytes and apoptosis in upregulated KEGG genes including multiple *PIK3* genes and NIK/NF-kappaB signaling and metabolic responses from lipopolysaccharides in downregulated KEGG genes including *CHUK* and *NFKB1*. Using FFPE samples, we determined DEPs characteristic of OSCC and distinct from BC. *KRT*-family genes and lipopolysaccharide producing periodontal pathogens may be further investigated for their involvement in the OPMD to OSCC transition.

## 1. Introduction

Oral squamous cell carcinomas (OSCCs) originate for the most part (~80%) from oral potentially malignant disorders (OPMDs). OPMDs have been characterized by lesions with a high risk of malignant transformation [1,2]. OPMDs include oral lichen planus, leukoplakia, proliferative verrucous leukoplakia, oral graft versus host disease, oral submucous fibrosis, and erythroplakia. Approximately 4.5% of the world’s population may have OPMDs with men being more frequently affected, likely due to increased use of tobacco and alcohol compared to women [3]. Other risk factors such as the use of betel nut derivatives, human papilloma virus (HPV) infection, oral microbiome dysbiosis, ill-fitting dentures, genetic alterations, compromised epigenetic regulation, dysregulated tumor microenvironment, or combinations of these factors may contribute to the progression of OPMDs to oral squamous cell carcinoma (OSCC) [4,5,6,7,8,9].

A common theme among OPMDs moving towards malignant transformation includes the loss of heterozygosity at chromosomal loci 3, 9, and/or 17 [10,11,12,13,14]. The loss of genetic material at these genomic regions implicates the early markers of oral carcinogenesis while loss of genetic material at chromosomes 8 and 13 are associated with late-stage carcinoma [15]. Furthermore, tissue markers such as *p53*, *EGFR*, *PD-L1*, CD4+, CD8+ T cell, *TLR-2*, *TNF-α*, *IL-6*, *COX-2*, *CD34*, *TGF-β*, and Mcm2 have been implicated for their involvement in OPMDs to cancer transition [9,11]. We have previously identified *Haemophilus pittmaniae* and *Leptotrichia* spp. as a multi-marker signature in a cohort of HPV positive head and neck cancer (HNC) patients. This finding suggests that oral bacterial species may coexist with HPV within HPV-induced oral lesions in HNC patients, contributing to the transition to OSCC [6].

Current treatments for OPMDs include the mitigation of risk factors and the removal of moderate to severe lesions and chemoprevention [8]. Less invasive approaches utilize anti-inflammatory drugs or topical medications [12,16]. The early screening of OPMDs is vital for timely diagnosis to minimize malignant transformation, yet procedures rely on the visual exploration of lesions. Furthermore, clinical approaches of suspicious lesions may include screening aids such as vital staining with toluidine blue and Lugol’s iodine, autofluorescence, chemiluminescence, narrow-band imaging, high-frequency ultrasounds, and biomarker assessment from saliva, serum, or exfoliated cells [12,17,18,19,20,21,22,23,24]. There is currently no methodology to predict the likelihood of transformation of an individual lesion to OSCC.

By discovering genetic biomarkers suitable to predict or pinpoint the stage of a lesion’s progression towards OSCC, new opportunities for the development of treatment strategies of OPMDs may arise. With the prospect of investigating OPMD lesions in future studies to generate a predictor algorithm, using the latest RNAseq technology, we first sought to determine transcriptomic profiles of oral lesions’ biopsies in patients that developed OSCC. Thus, we implemented an initial proof-of-concept pilot study of OSCC samples (*N* = 3). As an unrelated control group, we compared our OSCC data to transcriptomic RNAseq data obtained from the formalin-fixed paraffin-embedded samples of unrelated breast cancer biopsies (*N* = 6) and yielded OSCC relevant findings, thereby confirming that a proper methodology was used.

## 2. Results

**Figure 1:**
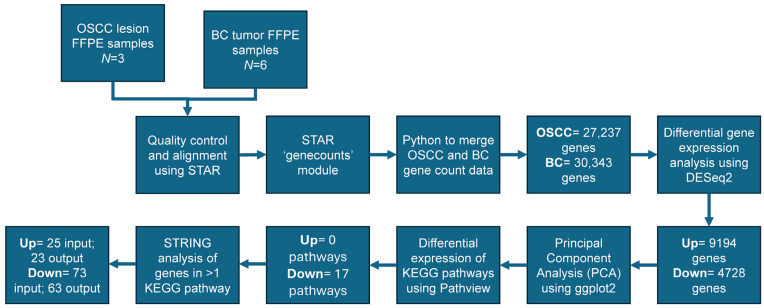
Analytical design: oral lesion formalin-fixed paraffin-embedded (FFPE) samples were collected from oral squamous cell carcinoma (OSCC) patients (*N* = 3) after the surveillance for oral potentially malignant disorders (OPMDs). OSCC FFPE samples (*N* = 3) were analyzed using RNAseq. Sequences from FFPE samples of OSCC patients (*N* = 3) (our data) were used in conjunction with breast cancer (BC) patients to perform a technical comparison (*N* = 6) (publicly available data from Gene Expression Omnibus (GEO) Database, GSE58135; https://www.ncbi.nlm.nih.gov/geo/query/acc.cgi?acc=GSE58135, accessed on 24 October 2024). Samples were aligned to the human reference (GRCh38.p13). Quality control was completed using FASTQC and genes were counted using Spliced Transcripts Alignment to a Reference (STARv2.7.9a) software. Count data was merged using pythonv3.12.4. Differential gene expression was determined in R using DESeq2v1.40.2 comparing OSCC (27,237 input genes) and BC (30,343 input genes) samples. A total of 9194 and 4728 genes were determined as up- or downregulated, respectively. Principal component analysis (PCA) was completed and plotted showing clear differentiation between OSCC and BC samples. Differential Kyoto Encyclopedia of Genes and Genomes (KEGG) pathways were determined via the Pathviewv.1.40.0 program determining 17 downregulated pathways. The Search Tool for the Retrieval of Interacting Genes (STRINGv12.0) was used to determine protein–protein interactions (PPIs) at the highest confidence level (CL = 0.900) between genes appearing in more than one KEGG pathway with an input of 25 upregulated and 73 downregulated genes returning 23 and 63 genes within the protein–protein interaction, respectively.

The demographic information for OSCC patients (*N* = 3) is presented in Table 1. The overall analytical pipeline of the study is presented in Figure 1. The pathological features of the three patients are shown in Supplemental Figure S1.

**Figure 2:**
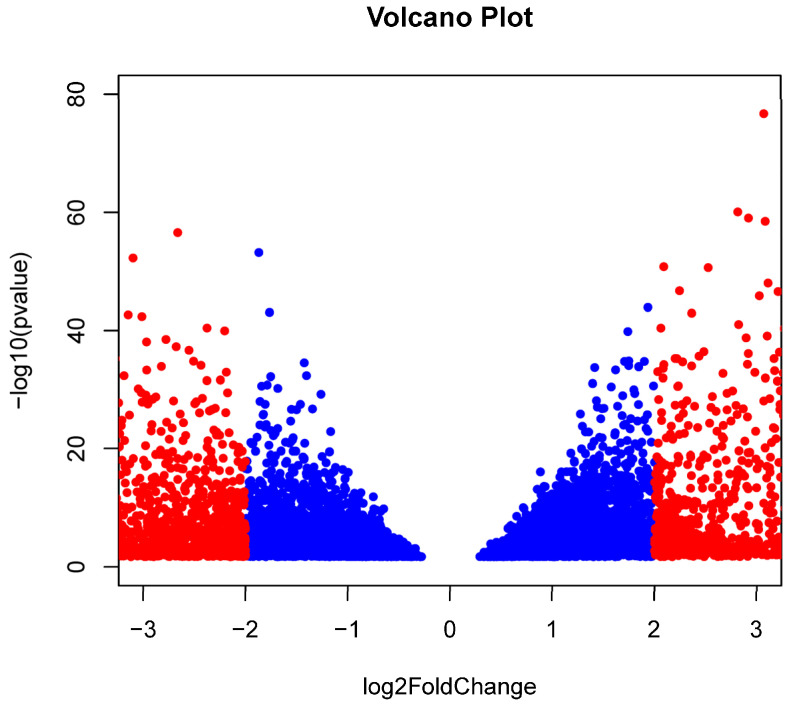
Differentially expressed genes: A volcano plot depicting the technical reference comparison of significant [*p* < −log10(0.05)] differentially expressed genes comparing formalin-fixed paraffin-embedded (FFPE) samples from lesions of oral squamous cell carcinoma (OSCC) patients (*N* = 3) and publicly available data investigating tumors of breast cancer (BC) patients (*N* = 6) determined using DESeq2v1.40.2 within Rv4.3.0. A total of 9194 genes were differentially expressed, with 4466 being upregulated (OSCC > BC; log_2_FoldChange > 0) and 4728 being downregulated (BC > OSCC; log_2_FoldChange < 0). Filtering results by restricting log_2_FoldChange as less than −2.0 and greater than 2.0 resulted in 3318 remaining genes with 1271 being upregulated (OSCC > BC) and 2047 being downregulated (BC > OSCC). Insignificant genes after filtering are shown in blue while significant genes for log_2_FoldChange are shown in red.

RNAseq for three OSCC and six BC formalin-fixed paraffin embedded (FFPE) samples were obtained at an average read depth of 16.2 and 43.3 million reads per sample with an average unique mapping of 82.44% and 79.75%, respectively. The STARv2.7.9a ‘genecounts’ module detected 27,237 and 30,343 genes among the OSCC and BC groups, respectively. DESeq2v1.40.2 determined that 9194 genes were differentially expressed, with 4466 being upregulated (OSCC > BC) and 4728 being downregulated (BC > OSCC) (padj < 0.05). Filtering results by restricting log_2_FoldChange (log_2_FC) to less than −2.0 and greater than 2.0 resulted in 3319 remaining genes with 1271 being upregulated (OSCC > BC) and 2048 being downregulated (BC > OSCC). A volcano plot showing significant genes is presented in Figure 2. Upregulated genes included *KRT6B*, *SERPINB5*, *DSC3*, and *PERP*, and *KRT5* (log_2_FC > 5.0; padj < 10^−200^) (Table 2a). Top downregulated genes included *KRT19*, *GREB1*, *ARFGEF3*, *SERPINA3*, *LONRF2* (log_2_FC < −4.0; padj < 10^−80^) (Table 2b). A list of all differentially expressed genes can be found in Supplemental File S1.

**Figure 3:**
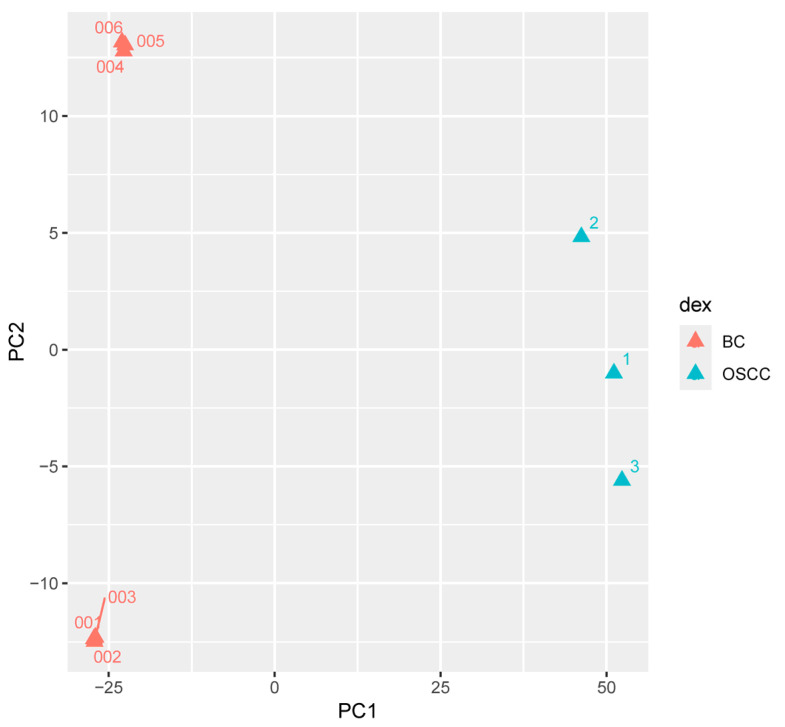
Principal component analysis comparing FFPE samples of lesions from OSCC patients to the tumors of BC patients; principal component analysis (PCA) of RNAseq gene counts comparing formalin-fixed paraffin-embedded (FFPE) FFPE samples from the lesions of patients that developed oral squamous cell carcinoma (OSCC; *N* = 3) and the FFPE samples of tumors taken from breast cancer (BC) patients (*N* = 6) reveals distinct grouping with the first principal component responsible for 87% variance between the OSCC and BC gene expression.

Principal Component Analysis (PCA) resulted in the first principal component being responsible for 87% of variance between OSCC and BC FFPE transcriptomic profiles. PCA also showed BC and OSCC samples clustered into distinct groups (Figure 3). Pathviewv.1.40.0 determined 17 downregulated Kyoto Encyclopedia of Gene and Genomes (KEGG) pathways in the OSCC group compared to the BC group. Pathways included chemokine signaling, natural killer cell mediated cytotoxicity, and NOD-like receptor signaling pathway. Pathviewv.1.40.0 was unable to identify any upregulated KEGG pathways. All 17 significant pathways and their involved genes are presented in Table 3. The Pathviewv.1.40.0 rendering of the top five most significant pathways can be found in Supplemental Figure S2a–e. The top significant pathways were ‘Chemokine signaling pathway’, ‘natural killer cell mediated cytotoxicity’, ‘NOD-like receptor signaling pathway’, ‘RIG-I-like receptor signaling pathway’, and ‘arginine and proline metabolism’.

**Figure 4:**
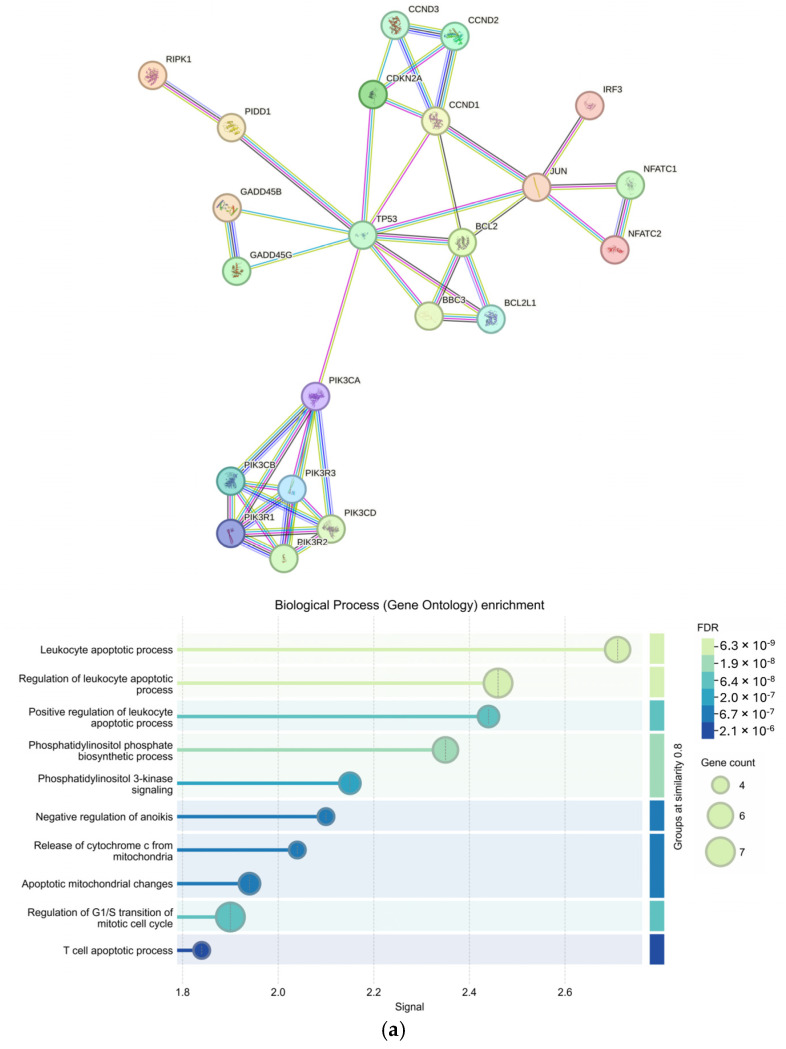

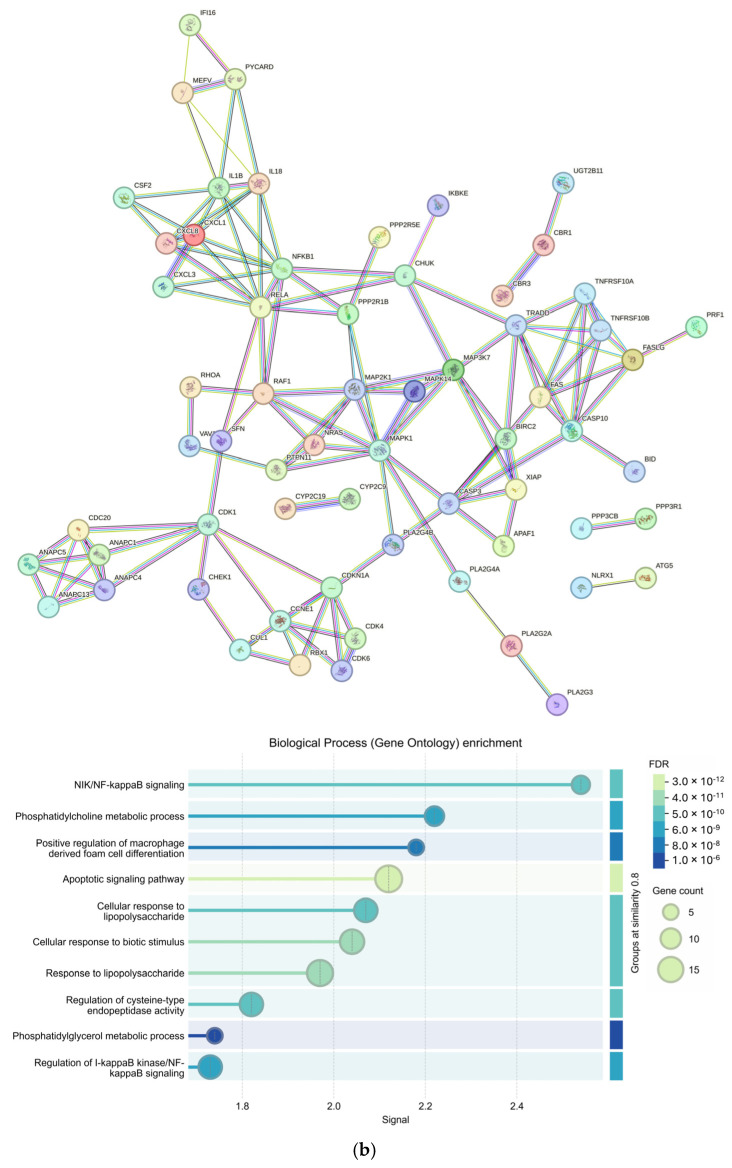
Protein–protein interactions of genes involved in more than one Kyoto Encyclopedia of Genes and Genomes (KEGG) pathway and their Gene Ontology Biological Process enrichment; Protein–protein interaction networks created using the Search Tool for the Retrieval of Interacting Genes/Proteins (STRINGv12.0) at the highest confidence (Conf = 0.900) and the corresponding Gene Ontology Biological Process (GO BP) enrichment for (**a**) upregulated (input = 25; output = 23) and (**b**) downregulated (input = 73; output = 63) genes appearing in more than one differential KEGG pathway (*p* < 0.05).

From an input of 25 upregulated genes appearing in more than one KEGG pathway, 23 genes were returned in the protein-protein interaction (PPI) by the Search Tool for the Retrieval of Interacting Genes (STRINGv12.0) (*p* < 1 × 10^−16^). Enriched Gene Ontology Biological Processes (GO BPs) included multiple terms related to leukocytes and apoptosis as well as cell regulation and signaling (FDR < 1 × 10^−5^) (Figure 4a). Furthermore, 63 genes were returned in the PPI from an input of 73 downregulated genes appearing in more than one KEGG pathway (*p* < 1 × 10^−16^). The top GO BP was determined to be NIK/NF-kappaB signaling (FDR = 1.19 × 10^−9^) with other significant GO BPs involving metabolic responses and signaling from external stimuli (Figure 4b).

## 3. Discussion

In this proof-of-concept pilot study, we were able to distinguish the transcriptomic profiles of OSCC patients’ oral lesions compared to BC tumors using RNA-seq of FFPE blocks based on data generated in our laboratory and data obtained from the Gene Expression Omnibus NCBI NIH database (GEO; https://www.ncbi.nlm.nih.gov/geo/query/acc.cgi?acc=GSE58135, accessed on 24 October 2024) that could be appropriately normalized for comparison. *KRT* genes have been associated with a multitude of cancers [25]. Our results showed *KRT* genes as the most significant up- and downregulated genes among the filtered gene set (Table 2). A total of eight *KRT* genes were found to be downregulated while 20 were found to be upregulated after filtering (Supplemental File S1). *KRT6B* was the most significantly upregulated gene among the OSCC group. Another type II cytokeratin, *KRT5*, has been previously associated with squamous cell carcinoma (SNP IDs rs11170164—chromosome 3:188370473 and rs607860) [26,27]. In our study, *KRT5* was determined as upregulated with *p* = 5.16 × 10^−236^ and a log_2_FC > 5.0 (Figure 2, Table 2). The type I cytokeratin, *KRT19*, was the most significantly downregulated gene (*p* = 3.07 × 10^−92^; log_2_FC < −9.0). KRT19 has been associated with the progression of dysplasia in leukoplakia [28]. *KRT* genes belong to the keratin gene family and are responsible for maintaining cellular integrity. A 2019 study identified *KRT31*, *37*, *76* as significantly different between leukoplakia groups with and without dysplasia [29]. No *KRT* genes were found in any differential KEGG pathways in this study (Table 3).

We identified 17 significant KEGG pathways involving genes expressed in our OSCC samples (Table 3). All significant pathways were downregulated when compared to BC samples. Pathways included chemokine signaling, natural killer cell mediated cytotoxicity, apoptosis, and others known to be involved in both BC and OSCC progression. *PIK3CB* was present in 4 of the 17 (~24%) pathways and PIK3 genes were tightly clustered within the upregulated STRINGv12.0 PPI network (Table 3 and Figure 4a). *PIK3CB* is a kinase important for signaling to receptors on the outer membranes of eukaryotic cells. *PIK3CB* can also activate neutrophils during injury or infection. Additionally, genes belonging to the PIK3 family of genes have been reported as the most frequently mutated oncogenes in human cancer [30]. The upregulation of *PIK3CB* in multiple pathways and the tight grouping of many PIK3 genes within the PPI network indicate a possible increase in cell proliferation, which is characteristic of the OPMD to OSCC transition and BC transformation [31,32]. The many connections of *CHUK* and *NFKB1* to other genes within the STRINGv12.0 PPI network of downregulated genes demonstrate this complex relationship (Figure 4b).

There may also be external factors influencing the immune response leading to the progression of BC or OSCC with significant GO BPs such as the ‘positive regulation of macrophage derived from cell differentiation’, ‘cellular response to lipopolysaccharide’ and ‘cellular response to lipopolysaccharide’. Lipopolysaccharides, produced by oral bacteria such as *Porphyromonas gingivalis*, *Prevotella intermedia*, and *Fusobacterium nucleatum* can activate macrophages leading to the production of inflammatory cytokines causing an immune response and tissue damage [33,34,35]. In OSCC, *P. gingivalis* has been shown to aid in the progression of cancer by (i) activating the expression of NF-kappaB and MAPK pathways, (ii) inhibiting apoptosis by activating jAk/stat and P13K/Akt, (iii) promoting angiogenesis by increasing expression of *EENB2*, (iv) increasing cell proliferation via *PDCD4* inhibition, increasing *AP1* and *CD1*, and (v) evading the host immune system through the production of butyric acid causing T-cell and B-cell apoptosis to ensure its survival on gingival epithelial cells [34]. The increased abundances of periodontal pathogens in OPMDs have been reported but more longitudinal studies at the species level are needed to clarify the mechanisms between the bacterial relationship and OPMD to OSCC transition and the potential for therapeutic interventions [36,37].

Although Pathviewv.1.40.0 and Gagev2.50.0-determined *CHUK* and *NFKB1* were downregulated in multiple pathways, DESeq2v1.40.2 analysis determined their expression to be 0.72 and 0.88 log_2_FC higher in OSCC samples than BC samples, respectively. This is likely due to Pathviewv.1.40.0 visualizing the net effect of multiple genes in a pathway. In other words, although *CHUK* and *NFKB1* are shown to be upregulated by DESeq2v1.40.2, an inhibitor of these genes upstream with higher expression may cancel out that effect, causing them to be visualized as downregulated in the pathway. *CHUK* and *NFKB1* are engaged in a complex crosstalk linked to cancer progression and drug resistance due to their ability to activate inflammatory responses and promote cell survival [38,39].

Although our analysis compared OSCC to BC samples, 19 significant genes overlapped with Farah and Fox’s 47 differentially expressed genes (DEGs), including the upregulation of *ODC1* and *LCN2* and the downregulation of *COL1A1*, *COL11A1,* and *STAC2* in dysplastic leukoplakia samples (Supplemental File S1) [29]. Furthermore, a study investigating the RNAseq of human tongue OSCCs vs. healthy tongue epithelia in the same 20 patients had 2543 overlapping genes with our DEGs prior to filtering [40]. Of the 2543 genes, 1397 (~55%) showed similar up (*n* = 780) /down (*n* = 617) regulation [40]. We also found 1554 DEGs in common (~38%) prior to filtering, with a 2015 study by Conway et al. comparing the FFPE sections of tumors from OSCC patients compared to the adjacent healthy tissues of 19 patients [41]. Over 25% of these genes also had similar up- (*n* = 126) and downregulation (*n* = 275). Despite these studies having significantly more samples than our study, we were able to confirm similar results, suggesting that the OSCC transcriptomic profiles are highly conserved across different patient populations.

### Limitations

The sample size in this pilot study is small. While we met the DESeq2v1.40.2 recommendation of having at least three samples per condition, a larger sample size would reduce the chance of false positives. While FFPE samples from breast cancer tumors functioned as a way to complete a technical reference comparison, comparing OSCC samples to healthy oral mucosa or premalignant lesions would be ideal; however, publicly available data from FFPE samples using Illumina for RNAseq are scarce. In future studies, we will compare the transcriptomic profiles of OPMD (early/pre-cancerous) lesions of FFPE samples from patients who developed OSCC to those who did not. The microbial profiles of patients in conjunction with transcriptomic profiles may be further investigated longitudinally to confirm the involvement of *P. gingivalis* and other periodontal pathogens. This would allow us to design an algorithm intended for the prediction of the OPMD to OSCC transition using transcriptomic and/or microbial shifts in patients.

## 4. Materials and Methods

### 4.1. Sample Collection and Patient Characteristics

Formalin-fixed and paraffin-embedded (FFPE) OSCC oral lesions samples were obtained from the Atrium Health Biospecimen Repository, Atrium Health, Charlotte, NC, USA associated with multiple clinical studies in which OSCC patients have consented for genomic analyses. The study was approved by the Wake Forest University Institutional Review Board (IRB00109068) and qualified for expedited review under the Federal Regulations [45CFR46.110]. Cases were identified by retrospective review. Patients (*N* = 3) who developed OSCC 3–5 years after initial presentation with OPMD oral lesions were identified through a patient record review and selected without a priori gender or race bias.

### 4.2. Initial Processing of FFPE Slides

Hematoxylin and eosin (H&E)-stained slides were independently reviewed by a single pathologist and classified in the low-grade dysplasia (LGD), moderate dysplasia (MD), high-grade dysplasia (HGD), squamous cell carcinoma (SCC), or no dysplasia/carcinoma categories, according to the most recent pathologic classification of oral cavity dysplasia by the World Health Organization (El-Naggar et al., 2022) [42]. The H&E slides with relevant findings (OSCC for the purposes of this study) were marked to indicate sections containing tumor for RNA extraction from unstained sections.

### 4.3. RNA Extraction

Each sample was delivered as 15 FFPE sections mounted on individual slides and an accompanying H&E slide. A number 11 scalpel blade with a number 3 handle was used to remove the paraffin surrounding the tissue area of interest. A new blade was then used to scrape the tissue of interest into a 1.5 mL microfuge tube. Five to fifteen slides were used for each sample. RNA was extracted using the Quick-RNA FFPE kit (Zymo Research, Irvine, CA, USA) and processed using the standard protocol except 2 volumes of 100% ethanol were used to increase the recovery of small fragment RNA. RNA was DNase-treated and purified using the RNA Clean and Concentrator-5 kit (Zymo Research, Irvine, CA, USA) and assessed for RNA quality using an Agilent 4200 TapeStation and the Standard RNA Assay Kit (Agilent Technologies, Santa Clara, CA, USA).

### 4.4. Bulk RNAseq Sequencing Method

Total RNA was used to prepare cDNA libraries using the Illumina^®^ TruSeq^®^ Stranded Total RNA Library Prep Globin (Illumina Inc., San Diego, CA, USA). RIN values for the RNA samples were quality assessed on an Agilent TapeStation (Agilent Technologies, Santa Clara, CA, USA). Briefly, 750 ng of total RNA was rRNA depleted followed by enzymatic fragmentation, reverse-transcription, and double-stranded cDNA purification using AMPure XP magnetic beads (Beckman Coulter, Inc., Brea, CA, USA). The cDNA was end repaired, 3′ adenylated, with Illumina sequencing adaptors ligated onto the fragment ends, and the stranded libraries were pre-amplified with PCR. The library size distribution was validated and quality inspected using an Agilent TapeStation (Agilent Technologies, Santa Clara, CA). The quantity of each cDNA library was measured using the Qubit 3.0 (Thermo Fisher Scientific, Waltham, MA, USA). The libraries were pooled and sequenced on the Illumina NextSeq2000, San Diego, CA, USA (or Illumina NovaSeq, San Diego, CA, USA).

### 4.5. Bioinformatics Analysis

Transcriptomic sequencing data for breast cancer samples to be used for technical comparison were obtained from the publicly available Gene Expression Omnibus (GEO; GSE58135; https://www.ncbi.nlm.nih.gov/geo/query/acc.cgi?acc=GSE58135, accessed on 24 October 2024) database for immunohistochemistry confirmed estrogen receptor-positive breast cancer FFPE samples (*N* = 6) (GSE58135) [43,44]. Adapters were trimmed from all samples (OSCC = 3; BC = 6) and aligned to the human reference genome (GRCh38.p13) using Spliced Transcripts Alignment to a Reference (STARv2.7.9a) [45]. The ‘genecounts’ module within STARv2.7.9a was utilized for counting genes [44]. Pythonv3.12.4 was used to merge the OSCC count data with BC count data. DESeq2v1.40.2 was used in Rv4.3.0 to compare FFPE gene counts of OSCCs to BC samples. PCA was completed on gene counts and plotted in Rv4.3.0 using ggplot2v3.5.1 to show the grouping of OSCC samples compared to BC samples.

The Gagev2.50.0 and Pathviewv.1.40.0 libraries were used within Rv4.3.0 to investigate the differential KEGG pathways [46]. STRINGv12.0 was used to determine PPIs of up- or downregulated genes appearing in more than one differential KEGG pathway at the highest confidence level and the corresponding GO BP enrichment [47].

## 5. Conclusions

There is a clear distinction in the transcriptomic profiles of FFPE samples in the lesions of patients that developed OSCC compared to the FFPE samples of breast cancer patients. Genes belonging to the *KRT* family may be further investigated for their involvement in the OPMD to OSCC transition.

## Figures and Tables

**Table 1:** OSCC demographic data.

Criteria ^a^	OSCC ^b^
Patient samples (M/F) ^c^	3 (2/1)
Patient race/ethnicity:	
Caucasian	3 (2/1)
Age ^d^:	
Median	70
Mean	69
Standard deviation	10.54
Range	58–79
Diagnosis ^e^:	
T4aN0	1 (1/0)
T1N0M0	1 (0/1)
T1N0Mx	1 (1/0)
Lesion type ^f^:	
Erythroleukoplakia	2 (1/1)
Leukoplakia	1 (1/0)

^a^ Demographic data for a cohort of formalin-fixed paraffin-embedded (FFPE) samples taken from patients diagnosed with ^b^ oral squamous cell carcinoma (OSCC); ^c^ total number of patients included in study. M is male, F is female; ^d^ Median, mean, standard deviation, and range of OSCC patient ages.; ^e^ OSCC diagnosis category; ^f^ Oral potentially malignant disorder (OPMD) lesion type from which sample was taken.

**Table 2:** Differentially expressed genes via DESeq2v1.40.2.

**(a)** Significantly Upregulated Genes with log_2_FoldChange > 2.0			
Gene ^a^	FC ^b^	*p*-Value ^c^	*p*-Adjusted ^d^
*KRT6B*	9.93	2.45 × 10^−276^	5.99 × 10^−272^
*SERPINB5*	6.37	7.95 × 10^−266^	9.72 × 10^−262^
*DSC3*	6.53	2.98 × 10^−251^	2.43 × 10^−247^
*PERP*	5.71	3.50 × 10^−244^	2.14 × 10^−240^
*KRT5*	5.34	1.06 × 10^−239^	5.16 × 10^−236^
*DSG1*	10.70	2.83 × 10^−208^	1.15 × 10^−204^
*DSC2*	6.27	1.75 × 10^−193^	6.10 × 10^−190^
*AQP3*	6.53	1.37 × 10^−153^	4.19 × 10^−150^
*GBP6*	9.06	1.37 × 10^−135^	3.71 × 10^−132^
*CLCA2*	7.54	6.75 × 10^−129^	1.65 × 10^−125^
*KRT14*	6.15	3.43 × 10^−120^	7.61 × 10^−117^
*TMPRSS11D*	5.65	2.82 × 10^−112^	5.74 × 10^−109^
*KLK7*	6.21	8.34 × 10^−111^	1.57 × 10^−107^
*DSG3*	8.16	2.30 × 10^−107^	4.02 × 10^−104^
*SPINK5*	8.96	1.23 × 10^−104^	2.00 × 10^−101^
*FABP5*	5.63	4.14 × 10^−101^	6.32 × 10^−98^
*LGALSL*	4.45	8.36 × 10^−100^	1.20 × 10^−96^
*DMKN*	4.74	2.56 × 10^−99^	3.47 × 10^−96^
*MPZL2*	4.06	3.00 × 10^−99^	3.85 × 10^−96^
*A2ML1*	8.01	6.88 × 10^−99^	8.41 × 10^−96^
*IVL*	11.55	1.30 × 10^−98^	1.51 × 10^−95^
*LIPG*	4.40	5.63 × 10^−98^	6.25 × 10^−95^
*PKP1*	6.33	7.62 × 10^−95^	7.75 × 10^−92^
*GLTP*	3.97	1.55 × 10^−92^	1.46 × 10^−89^
*PLA2G4E*	10.23	2.62 × 10^−92^	2.37 × 10^−89^
*KRT17*	5.40	1.86 × 10^−90^	1.63 × 10^−87^
*CERS3*	7.25	3.77 × 10^−90^	3.18 × 10^−87^
*BNC1*	5.71	2.08 × 10^−89^	1.70 × 10^−86^
*TRIM29*	3.90	2.70 × 10^−86^	2.06 × 10^−83^
*KLK5*	4.74	1.97 × 10^−85^	1.46 × 10^−82^
*FAT2*	4.41	4.13 × 10^−85^	2.97 × 10^−82^
*TRPV3*	5.74	1.46 × 10^−79^	1.02 × 10^−76^
*CNFN*	6.47	2.78 × 10^−78^	1.89 × 10^−75^
*LYPD5*	5.20	1.29 × 10^−77^	8.54 × 10^−75^
*COL17A1*	3.07	1.89 × 10^−77^	1.21 × 10^−74^
*IL20RB*	6.40	7.89 × 10^−76^	4.82 × 10^−73^
*SERPINB13*	12.73	1.12 × 10^−75^	6.68 × 10^−73^
*CALML3*	5.59	1.67 × 10^−75^	9.50 × 10^−73^
*SFN*	5.65	1.07 × 10^−71^	5.69 × 10^−69^
*ARNTL2*	4.52	8.59 × 10^−71^	4.28 × 10^−68^
**(b)** Significantly downregulated genes with Log_2_FoldChange < −2.0			
Gene ^a^	FC ^b^	*p*-value ^c^	*p*-adjusted ^d^
*KRT19*	−9.03	2.89 × 10^−95^	3.07 × 10^−92^
*GREB1*	−6.64	3.99 × 10^−94^	3.90 × 10^−91^
*ARFGEF3*	−4.36	1.53 × 10^−88^	1.21 × 10^−85^
*SERPINA3*	−5.37	2.54 × 10^−77^	1.59 × 10^−74^
*LONRF2*	−6.77	1.31 × 10^−75^	7.61 × 10^−73^
*PLEKHA6*	−3.90	8.37 × 10^−74^	4.65 × 10^−71^
*AR*	−4.80	7.66 × 10^−73^	4.16 × 10^−70^
*APOD*	−5.36	1.16 × 10^−71^	6.02 × 10^−69^
*EGR3*	−4.58	1.38 × 10^−71^	7.02 × 10^−69^
*CRACR2B*	−6.19	2.76 × 10^−70^	1.35 × 10^−67^
*ESR1*	−3.81	4.83 × 10^−69^	2.23 × 10^−66^
*HID1*	−3.76	8.83 × 10^−66^	3.92 × 10^−63^
*PCLO*	−6.35	9.72 × 10^−66^	4.24 × 10^−63^
*EFHD1*	−6.48	5.46 × 10^−62^	2.19 × 10^−59^
*PRLR*	−6.61	2.06 × 10^−61^	7.88 × 10^−59^
*KRT8*	−6.80	6.01 × 10^−59^	2.13 × 10^−56^
*TFAP2B*	−6.23	9.49 × 10^−59^	3.31 × 10^−56^
*SNORA22*	−6.27	4.64 × 10^−58^	1.60 × 10^−55^
*CCN2*	−2.66	2.77 × 10^−57^	9.39 × 10^−55^
*TRNH*	−4.15	8.18 × 10^−57^	2.70 × 10^−54^
*MLPH*	−5.67	6.93 × 10^−56^	2.20 × 10^−53^
*FOXA1*	−8.03	1.84 × 10^−54^	5.61 × 10^−52^
*RHPN1*	−4.44	4.51 × 10^−54^	1.36 × 10^−51^
*SYCP2*	−3.10	5.13 × 10^−53^	1.46 × 10^−50^
*MGP*	−3.78	8.97 × 10^−53^	2.46 × 10^−50^
*PLIN4*	−3.74	8.90 × 10^−53^	2.46 × 10^−50^
*PNPLA2*	−3.31	1.30 × 10^−51^	3.45 × 10^−49^
*PLEKHS1*	−5.06	9.53 × 10^−51^	2.40 × 10^−48^
*KRT18*	−4.03	2.99 × 10^−50^	7.16 × 10^−48^
*SNORD15B*	−4.04	9.10 × 10^−50^	2.14 × 10^−47^
*CILP*	−6.10	2.34 × 10^−48^	5.34 × 10^−46^
*PADI2*	−3.47	7.78 × 10^−48^	1.73 × 10^−45^
*ELAPOR1*	−6.84	1.65 × 10^−47^	3.60 × 10^−45^
*WNK2*	−5.32	2.66 × 10^−47^	5.65 × 10^−45^
*CNN1*	−5.38	5.24 × 10^−47^	1.10 × 10^−44^
*GATA3*	−6.73	3.24 × 10^−46^	6.49 × 10^−44^
*HSPB6*	−7.48	8.88 × 10^−45^	1.71 × 10^−42^
*OXTR*	−6.85	2.66 × 10^−44^	4.99 × 10^−42^
*TRNR*	−3.54	4.44 × 10^−44^	8.22 × 10^−42^
*C3*	−3.72	8.26 × 10^−44^	1.51 × 10^−41^

Footnote. The top significantly (*p*-adjusted < 0.05) expressed genes. Results were filtered to only include genes with log_2_foldchanges a. upregulated greater than 2.0 or b. downregulated less than -2.0. Genes determined using DESeq2v1.40.2 comparing formalin-fixed paraffin-embedded (FFPE) samples of lesions from patients with oral squamous cell carcinoma (OSCC; *N* = 3) to tumors of breast cancer (BC) patients (*N* = 6); a Entrez gene symbol; b Log_2_FoldChange value of the gene determined using DESeq2v1.40.2 in Rv4.3.0; c *p*-value via the Wald test using DESeq2v1.40.2 in Rv4.3.0; d Benjamini–Hochberg adjusted *p*-values determined using DESeq2v1.40.2 in Rv4.3.0.

**Table 3:** Significantly downregulated KEGG pathways.

Pathway ^a^	KEGG ID ^b^	*p*-Value ^c^	Upregulated Genes ^d^	Downregulated Genes ^e^
Chemokine signaling pathway	hsa04062	0.0014	*ADCY1, ADCY2, ADCY3, ADCY5, ADCY7, ARRB1, ARRB2, GRK2, GRK5, PIK3CA, **PIK3CB**, **PIK3CD**, **PIK3R1**, **PIK3R2**, **PIK3R3**, PLCB4, **PLCG2**, PREX1, PXN*	*CCL13, **CCL2**, CCL20, CCL21, CCL22, CCL28, CCL8, CCR2, CCR8, **CHUK**, CX3CL1, **CXCL1**, CXCL14, **CXCL3**, CXCL5, CXCL6, **CXCL8**, CXCR1, CXCR4, CXCR6, GNAI1, GNAI2, GNAI3, GNB3, GNB5, GNG12, GNG7, HCK, JAK2, LYN, **MAP2K1**, **MAPK1**, **NFKB1**, PARD3, **RAF1**, RAP1A, **RHOA**, ROCK2, **SHC2**, **SHC3**, STAT1, STAT3, TIAM1, **VAV1***
Natural killer cell mediated cytotoxicity	hsa04650	0.0025	*ARAF, **IFNAR1**, **IFNAR2**, IFNGR1, IFNGR2, **NFATC1**, **NFATC2**, **PIK3CB**, **PIK3R3**, **RAF1**, TYROBP*	***CASP3*** *, **CSF2**, **FAS**, **FASLG**, FCGR3B, **MAP2K1**, **NRAS**, **PLCG2**, **PPP3CB**, **PPP3R1**, **PRF1**, PRKCA, **PTPN11**, RAET1E, RAET1L, SH3BP2, SHC1, **SHC2**, **SHC3**, SYK, ULBP3, **VAV1***
Arachidonic acid metabolism	hsa00590	0.0028	*CYP2B7P, GPX4, PTGDS, PTGR2, ZADH2*	*ALOXE3, **CBR1**, **CBR3,** CYP2C1, **CYP2219**, **CYP2C9**, CYP4F3, HPGD, LTA4H, **PLA2G2A**, **PLA2G2F**, **PLA2G3**, **PLA2G4A**, **PLA2G4B**, PLA2G4D, **PLA2G5**, **PLA2G6**, **PLAAT2, PLAAT3**, PTGES3, PTGS1*
NOD-like receptor signaling pathway	hsa04621	0.0045	***BCL2, BCL2L1*** *, BRCC3, CARD9, **CCL2**, **IRF3**, **JUN**, MAP1LC3B, **MAPK10**, MAVS, PLCB1, PRKCD, RBCK1, SHARPIN, TAB1, TAB3, TICAM1, TP53BP1, TRAF5*	***ATG5*** *, **BIRC2**, CARD16, CARD18, CARD6, CASP1, CASP4, CASP5, **CHUK**, CTSB, **CXCL1, CXCL3**, **CXCL8**, DEFB4B, DHX33, DNM1L, GBP5, **IFI16**, **IFNAR1**, **IFNAR2**, **IL18**, **IL1B**, IRAK4, MAPK13, **MAPK14**, MCU, **MEFV**, MFN1, MFN2, MYD88, NEK7, **NFKB1**, NLRP1, NLRX1, NOD2, PSTPIP1, **PYCARD**, **RELA**, **RHOA**, RNASEL, STAT2, SUGT1, TBK1, TLR4, TRAF3, TRPV2, TXN2, VDAC1, VDAC2*
RIG-I-like receptor signaling pathway	hsa04622	0.0123	***IRF3*** *, **RIPK1**, TBKBP1, TKFC*	***ATG5*** *, **CASP10**, **CHUK**, CYLD, IFIH1, IFNE, IFNK, **IKBKE**, **MAP3K7**, **NFKB1**, NLRX1, **TRADD**, TRIM25*
Arginine and proline metabolism	hsa00330	0.0144	*CARNS1, GAMT, MAOA, PYCR1, SMS, SRM*	*ALDH1B1, ALDH2, ALDH3A2, ALDH9A1, AOC1, ARG1, CNDP2, GOT2, NOS1, ODC1, P4HA2, SMOX*
Cell cycle	hsa04110	0.0245	*ABL1, ATRX, **CCND1**, **CCND2**, **CCND3**, CCNH, **CDKN2A**, CREBBP, DBF4B, E2F4, E2F5, **GADD45B**, **GADD45G**, MAU2, MYC, SMC1A, TGFB2, TGFB3, **TP53***	***ANAPC1, ANAPC13,*** ***ANAPC4**, **ANAPC5**, ATM, ATR, BUB1B, CCNA2, **CCNE1**, CDC14A, CDC14B, **CDC20**, CDC25B, CDC27, CDC6, CDCA5, **CDK1, CDK4, CDK6**, **CDKN1A**, CDKN2B, **CHEK1**, **CUL1**, ESPL1, FBXO5, KNL1, MAD2L1, MAD2L1BP, MCM4, MCM6, NDC80, ORC1, PPP2CA, PPP2R1A, PPP2R1B, PPP2R5C, **PPP2R5E**, PRKDC, PTTG1, RB1, RBX1, **SFN**, SGO1, STAG1, STAG2, TFDP1, TFDP2, YWHAG, YWHAQ, YWHAZ *
Apoptosis	hsa04210	0.0248	***BBC3*** *, **BCL2, BCL2L1**, FOS, **GADD45G**, **JUN**, **MAPK10**, PARP3, **PIDD1**, **PIK3CA**, **PIK3CB**, **PIK3CD**, **PIK3R1**, **PIK3R2**, SEPTIN4, **TP53**, TUBA1A, TUBA1C, TUBA3D, TUBA3E, TUBA4A, TUBA8*	***APAF1*** *, BAK1, BCL2A1. **BID**, **BIRC2**, CAPN2, **CASP10**, **CASP3**, CASP7, **CHUK**, CSF2RB, CTSC, CTSD, CTSH, CTSV, EIF2S1, **FAS**, **FASLG**, KRAS, MAP3K5, MCL1, **NFKB1**, **NRAS**, **PRF1**, **RAF1**, **RELA**, **TNFRSF10A**, **TNFRSF10B**, **TRADD**, **XIAP***
Cytosolic DNA-sensing pathway	hsa04623	0.0253	*DNASE2, **IRF3**, POLR2F, POLR3A, POLR3B, POLR3G, POLR3GL, **RIPK1***	***CASP3*** *, CGAS, **CHUK**, G3BP1, GSDME, **IFI16**, **IKBKE**, **IL18**, **IL1B**, **MEFV**, MLKL, **NFKB1**, **PYCARD**, **RELA**, SAMHD1*
Ubiquitin-mediated proteolysis	hsa04120	0.0340	*KLHL9, UBE2Q2, UBE2QL1*	***ANAPC1*** *, ANAPC10, **ANAPC13**, **ANAPC4**, **ANAPC5**, **BIRC2**, BIRC6, BRCA1, CBL, CBLC, **CDC20**, COP1, **CUL1**, CUL5, FBXW11, FBXW7, HERC4, MID1, NEDD4L, **RBX1**, SAE1, SKP2, TRIP12, UBA2, UBC, UBE2B, UBE2D1, UBE2D2, UBE2D3, UBE2E3, UBE2F, UBE2G1, UBE2K, UBE2N, UBE4A, **XIAP***
p53 signaling pathway	hsa04115	0.0361	***BBC3*** *, **BCL2, BCL2L1**, **CCND1**, **CCND2, CCND3**, **CDKN2A**, **GADD45B**, **GADD45G**, IGF1, **PIDD1**, TEAP3, THBS1, **TP53**, TSC2*	***APAF1*** *, **BID**, **CASP3**, CCNB1, CCNB2, **CCNE1**, **CDK1**, **CDK6**, **CDKN1A**, **CHEK1**, CYCS, EI24, **FAS**, GORAB, GTSE1, PERP, RRM2, RRM2B, SERPINB5, SESN3, **SFN**, SHISA5, SIAH1, **TNFRSF10A**, **TNFRSF10B**, TP53AIP1, ZMAT3*
Proteasome	hsa03050	0.0387	*ADRM1, PSMC1, PSMF1*	*PSMA1, PSMA2, PSMA4, PSMA5, PSMA6, PSMA7, PSMB1, PSMB2, PSMB5, PSMB6, PSMB7, PSMC3, PSMC6, PSMD1, PSMD12, PSMD14, PSMD2, PSMD6, PSMD9, PSME4, SEM1*
Retinol metabolism	hsa00830	0.0390	***CYP2A6*** *, **CYP2A7**, **CYP3A5***	***ADH1B*** *, **ADH5**, **ADH7**, CYP26B1, CYP27C1, **CYP2A6, CYP2A7**, CYP2C18, **CYP2C9**, **CYP2S1**, DHRS3, DHRS9, RDH10, RDH11, RDH12, RDH16, RPE65, SDR16C5, **UGT2B11***
Linoleic acid metabolism	hsa00591	0.0394		***PLA2G2A*** *, **PLA2G5**, **PLA2G2F**, **PLA2G4A**, CYP2C19, **PLAAT2, PLAAT3**, **PLA2G4B**, PLA2G4E, PLA2G4F, **PLA2G3**, **PLA2G6***
Metabolism of xenobiotics by cytochrome P450	hsa00980	0.0464	*AKR7A3, AKR7L, CYP1B1, **CYP2A6**, **CYP2A7**, **CYP2S1**, EPHX1, **GSTA4**, **GSTM4**, **GSTO1**, MGST2, **MGST3***	***ADH1B*** *, **ADH5**, **ADH7**, ALDH3A1, **CBR1**, **CBR3**, **CYP2C9**, **CYP3A5**, **GSTA4**, GSTM2, **GSTM4**, **GSTO1**, GSTP1, HSD11B1, MGST1, **MGST3**, **UGT2B11***
T cell receptor signaling pathway	hsa04660	0.0499	***JUN*** *, MAP2K7, **MAPK10**, **NFATC1**, **NFATC2**, **PIK3CB**, **PIK3R1**, **PIK3R2**, **PIK3R3***	*CDC42, **CDK4**, **CHUK**, **CSF2**, ICOS, **MAP2K1**, **MAP3K7**, **MAPK1**, MAPK11, **MAPK14**, NCK1, **NFKB1**, NFKBIE, **NRAS**, PPP2CB, **PPP2R1B**, PPP2R2A, PPP2R2C, PPP2R3A, **PPP2R5E**, **PPP3CB**, **PPP3R1**, **PTPN11**, PTPRC, **RAF1**, RASGRP1, **RELA**, **RHOA**, **VAV1***

^a^ Kyoto Encyclopedia of Genes and Genomes (KEGG) pathway name; ^b^ KEGG pathway ID; ^c^ FDR-corrected *p*-value determined via two-sided *t*-test using the; ^d^ Entrez gene symbols of differentially expressed genes (DEGs) upregulated in each pathway; ^e^ Entrez gene symbols of DEGs downregulated in each pathway; Note: genes shown in **bold** are present in more than one pathway.

## Data Availability

Data is contained within the article and Supplementary Material. Additional information for RNAseq processing can be found on our lab’s Github page (www.github.com/mbeckm01/FFPE_RNAseq).

## Supplementary Materials

The following supporting information can be downloaded at: https://www.mdpi.com/article/10.3390/ijms26136263/s1.
