# Supplementary material for: Transcriptomic Profile of Oral Cancer Lesions: A Proof-of-Concept Pilot Study of FFPE Tissue Sections
Source: Int J Mol Sci. 2025 Jun 28;26(13):6263. doi: 10.3390/ijms26136263 (PMC12250292; doi:10.3390/ijms26136263)
Supplement: Supplementary file 1 [file ijms-26-06263-s001.zip › Supplemental Figure S1. OSCC lesion pathology 04 11 2025 JLM FINAL.pdf]

Supplemental Figure S1. OSCC lesions sampled per patient

a. **Patient #1 lesion**

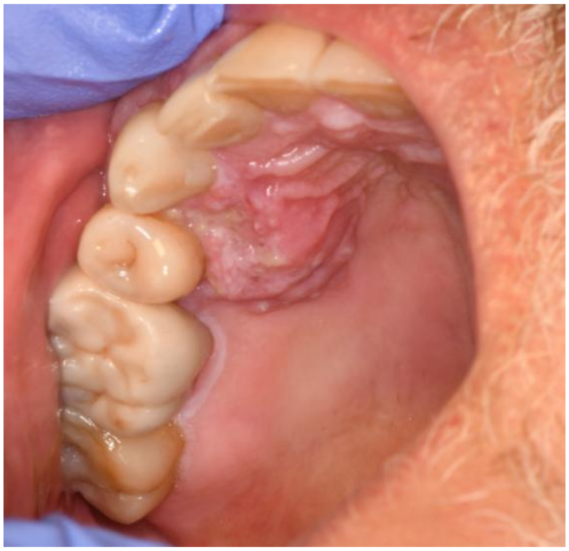

b. **Patient #2 lesion**

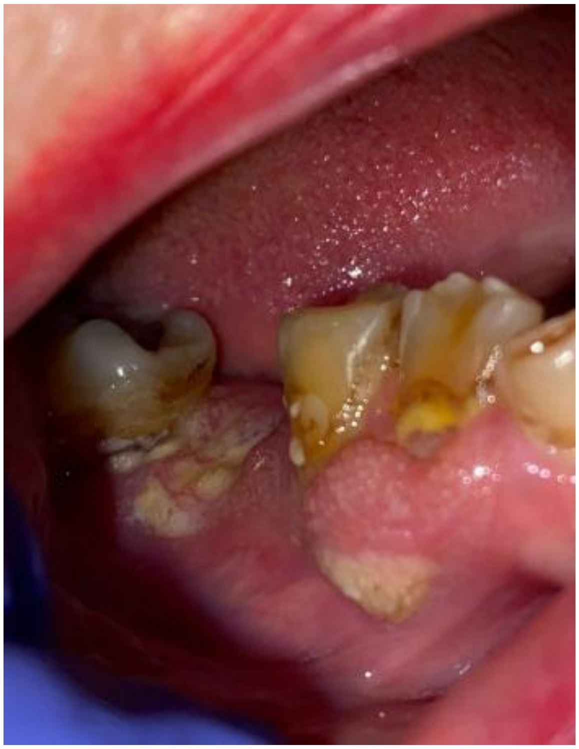

c. Patient #3 lesion

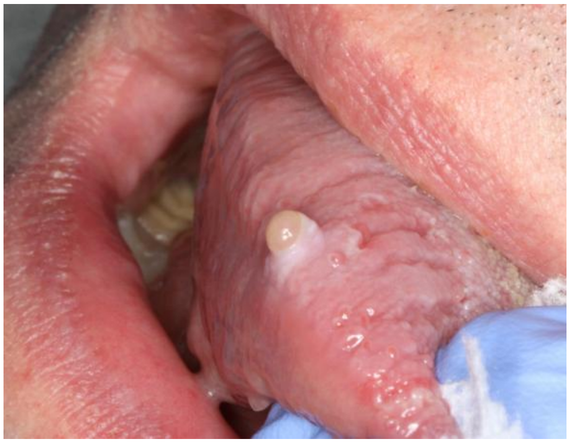

Legend. Formalin-fixed paraffin-embedded (FFPE) samples were collected from oral OSCC lesions in three patients following potentially malignant disorder (OPMD) surveillance over 3-5 years. Transcriptomic profiles were then determined using RNASeq.
