# Supplementary material for: Transcriptomic Profile of Oral Cancer Lesions: A Proof-of-Concept Pilot Study of FFPE Tissue Sections
Source: Int J Mol Sci. 2025 Jun 28;26(13):6263. doi: 10.3390/ijms26136263 (PMC12250292; doi:10.3390/ijms26136263)
Supplement: Supplementary file 1 [file ijms-26-06263-s001.zip › Supplemental Figure S2.a-e RNASeq FFPE OSCC Top 5 KEGG pathways-MB FBM JLM 03 06 2025.pdf]

Supplemental Figure S2. Top 5 significantly downregulated KEGG pathways

a. **Chemokine signaling pathway (p=0.0014)**

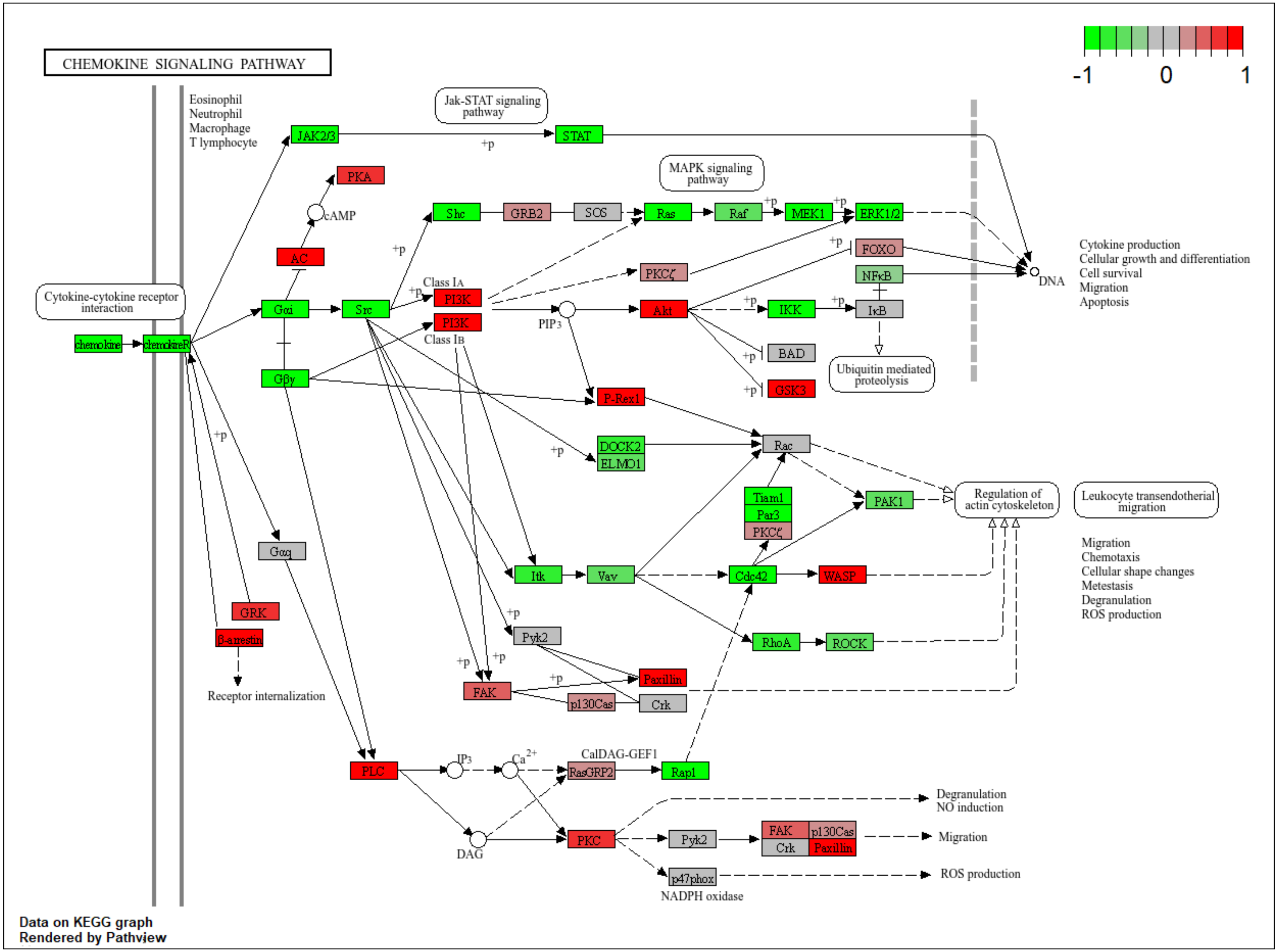

b. Natural killer cell mediated cytotoxicity (p=0.0025)

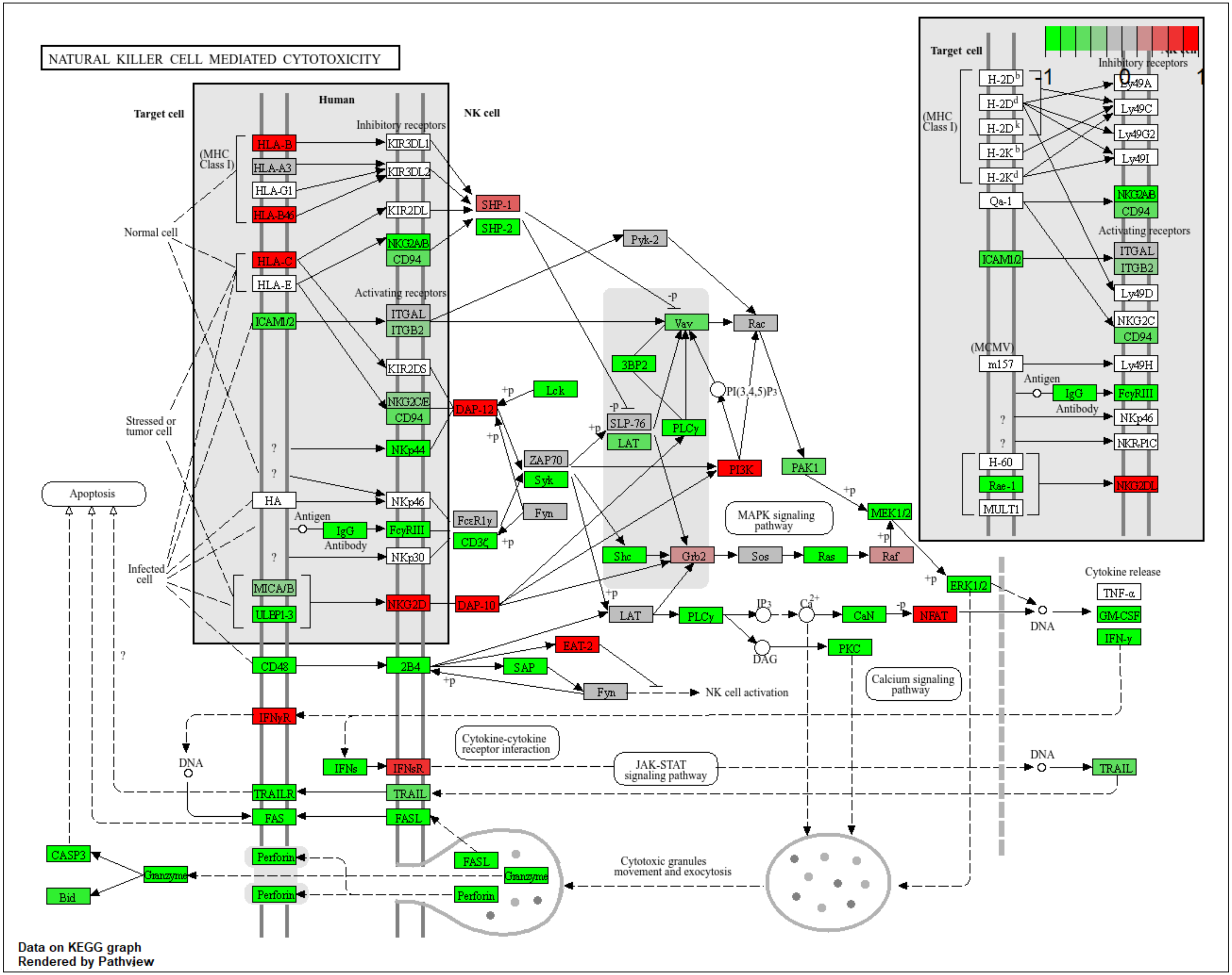

c. NOD-like receptor signaling pathway (p=0.0045)

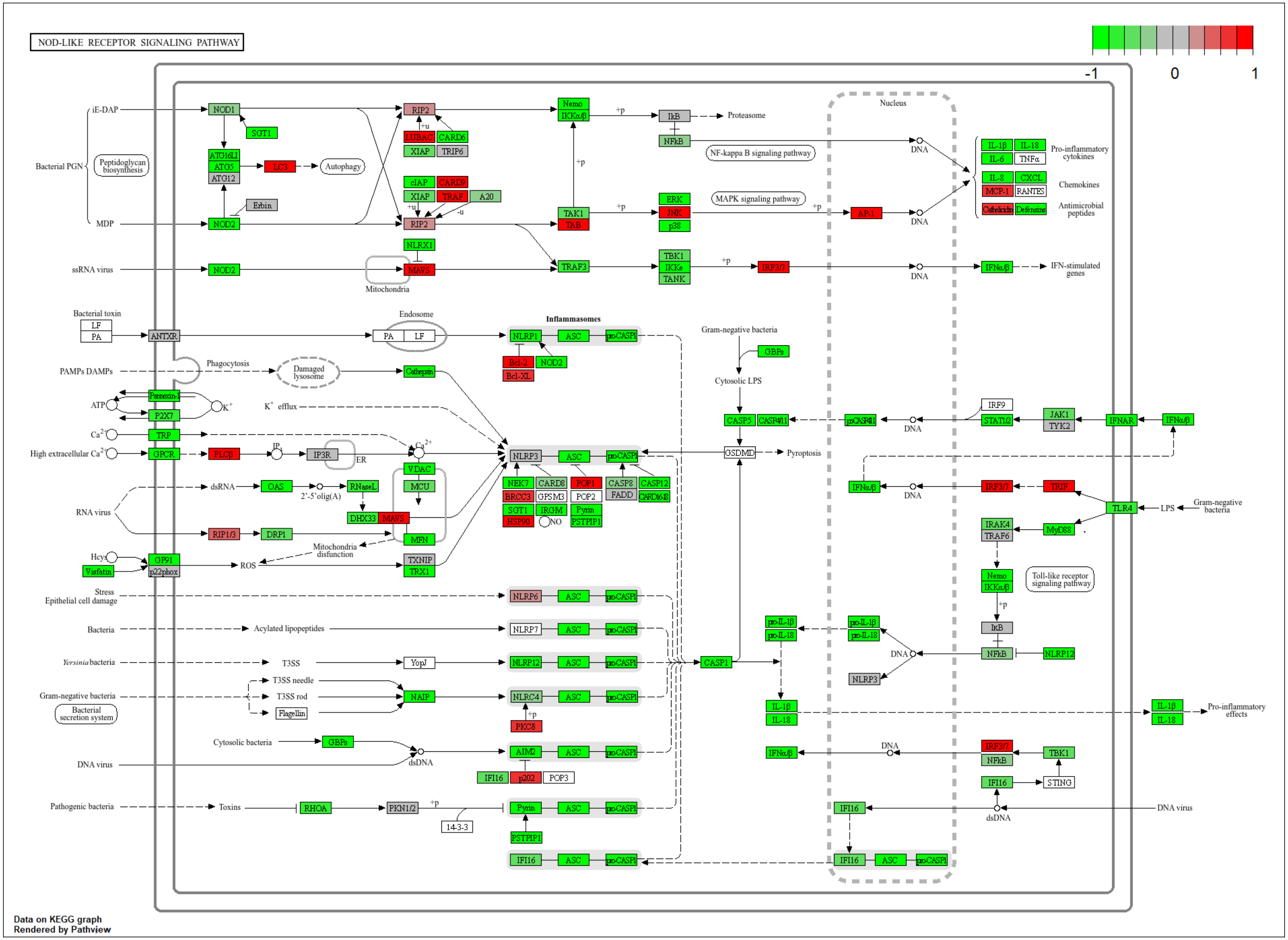

d. RIG-I-like receptor signaling pathway (p=0.012)

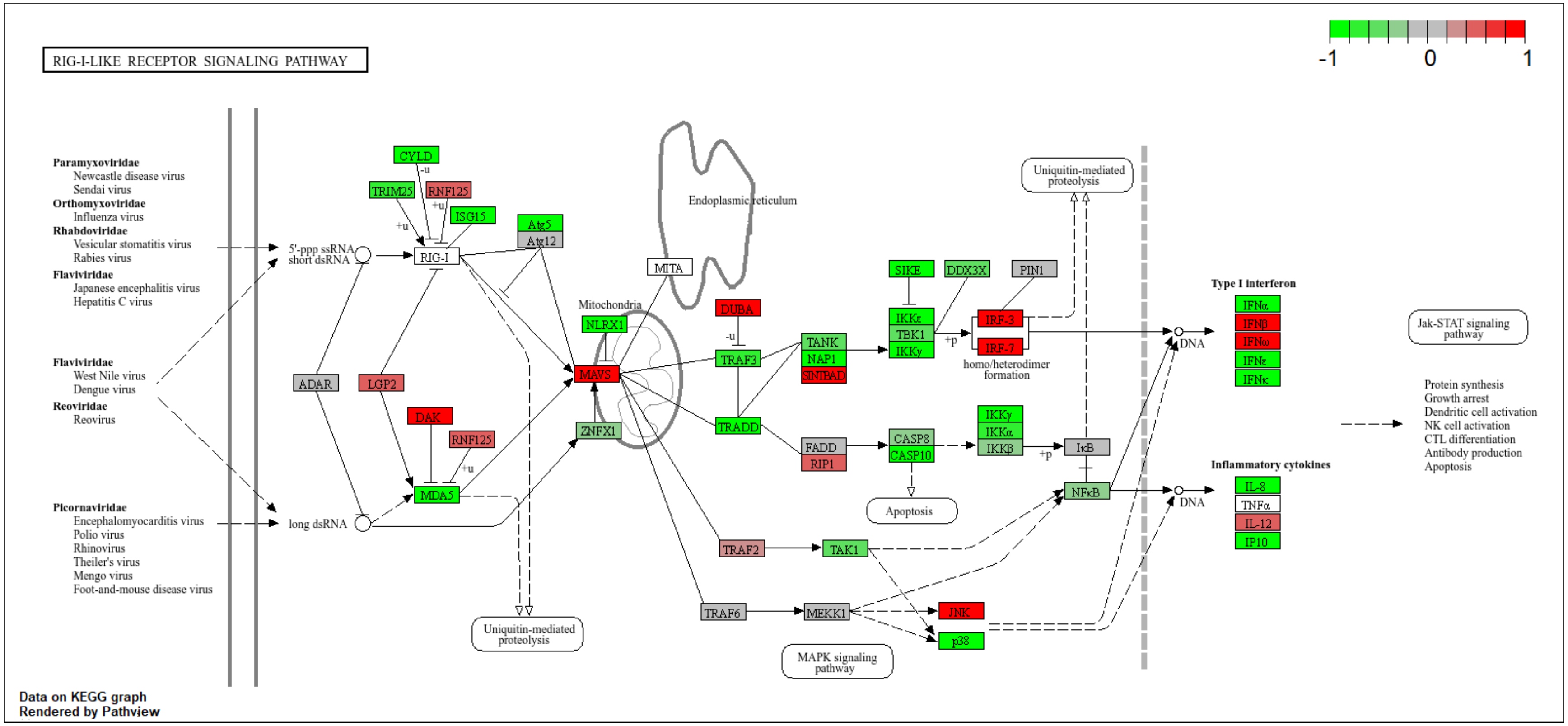

e. Arginine and proline metabolism (p=0.014)

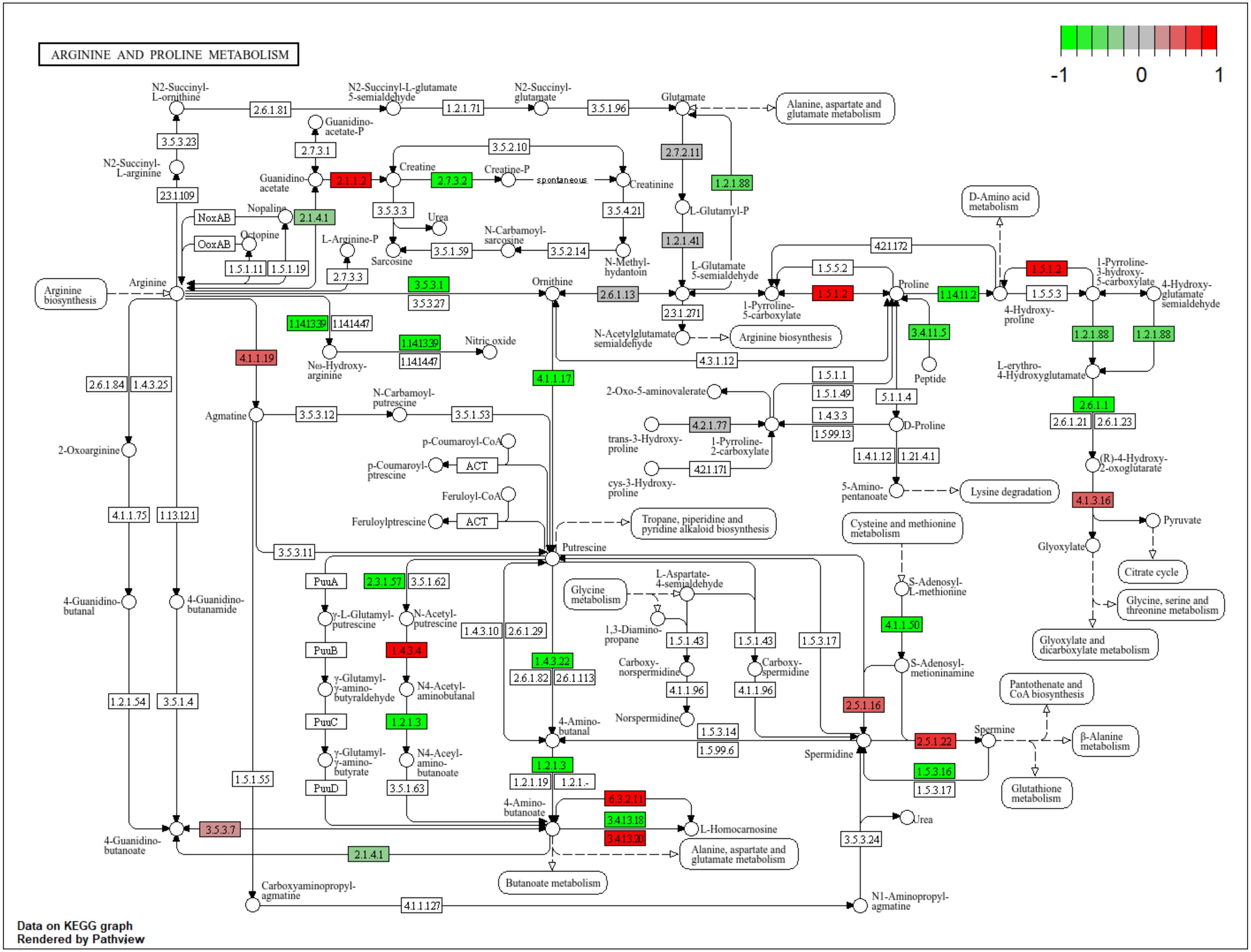

Legend. The top 5 of 17 total significantly downregulated KEGG pathways ($p<0.05$) rendered by Pathview in R showing downregulated (green) genes and upregulated (red) genes from our gene set as determined by the Gage and Pathview R libraries, comparing FFPE samples of lesions from patients that developed OSCC and tumors of breast cancer patients.
